# Children and Adolescents with Chronic Myeloproliferative Neoplasms: Still an Unmet Biological and Clinical Need?

**DOI:** 10.1097/HS9.0000000000000283

**Published:** 2019-08-01

**Authors:** Tariq I. Mughal, Michael W. Deininger, Nicole Kucine, Giuseppe Saglio, Richard A. Van Etten

**Affiliations:** 1 Tufts University Cancer Center, Boston, MA, USA; 2 University of Utah, Salt Lake City, UT, USA; 3 Weill Cornell Medicine, New York, NY; 4 Orbassano University Hospital, Turin, Italy; 5 University of California Irvine, Irvine, CA, USA.

The remarkable story of the classic chronic myeloproliferative neoplasms (MPN), a group of clonal hematological malignancies characterized by excessive accumulation of one or more myeloid cell lineages and an inherent ability to transform to acute leukemia, and views on its molecular genetics, biology and treatment continue to evolve.^1,2^ These neoplasms comprise of chronic myeloid leukemia (CML), defined by the presence of *BCR-ABL1*, and the *BCR-ABL1*-negative disorders, polycythemia vera (PV), essential thrombocythemia (ET) and primary myelofibrosis (PMF). The term myelofibrosis (MF) includes primary myelofibrosis (PMF) and post-PV and post-ET MF. Though the diagnostic criteria and classification in accordance to the revised 2016 WHO MPN criteria are often utilized in adults, adolescents and children, there are emerging data pointing to genetic differences which impact phenotypic characteristics, treatment and prognosis.^3,4^ In addition, most of the current risk scores used for adults have not been validated in children and adolescents, with the sole exception of the EUTOS long term survival score (ELTS) that may be used to predict progression-free survival, but not overall survival (OS).^5,6^ At the thirteenth post-American Society of Hematology (ASH) Workshop on CML and MPNs, which took place on the 4th to 5th December 2018 in San Diego, some of the unique biology, emerging age-related genomic features and treatment challenges pertaining to childhood and adolescents MPN were reviewed, with updated discussions since. Here, we briefly summarize these findings and indicate where these may have important clinical management implications; broader aspects of the workshop are published elsewhere.^7^

## Epidemiology and genetics

With the sole exception of juvenile myelomonocytic leukemia (JMML) and possibly cutaneous mastocytosis, the incidence of all MPNs increases with age.^8^ This observation lends support to the potential link between aging and inflammation (‘inflammaging’) as a driver of clonal evolution and selection in MPNs.^9^ MPNs are extremely rare in children and adolescents, and even rarer in infants. Also, unlike in adult MPNs, clonal hematopoiesis of indeterminate potential (CHIP) is unlikely in pediatric populations, and hence unlikely to play a role in the pathogenesis of pediatric MPNs.^10,11^ That said, there is some evidence suggestive of the notion of colonization of hematopoietic tissues by intestinal microbia being associated with the development of MPN in *TET2* deficient mice, a process mediated by IL-6 and linking progression from CHIP to MPNs to extrinsic ‘macro-environmental’ factors.^12–14^ Such observations lend some support to the hypothesis supporting the risk of a history of infection or autoimmune disease and myeloid malignancies.^15^

Children and adolescents with CML do not appear to have cytogenetic abnormities in addition to the Philadelphia chromosome, but have been observed to harbor different *BCR-ABL1* breakpoint distribution and additional somatic mutations, often resembling those seen in *BCR-ABL1*-positive acute lymphoblastic leukemia (ALL).^16,17^ The leukemogenesis of pediatric *BCR-ABL1*-negative MPNs is associated with genetic drivers that are similar to those in adults, but with important differences. For example, a lower frequency of mutations in all 3 founding driver mutations, *JAK2, MPL* and *CALR,* and an absence of mutations in any of the about 100 known MPN-associated genes have been reported in a significant proportion of children and adolescents diagnosed with MPNs.^18–20^ It is of interest that many children without these mutations have a diagnosis of hereditary thrombocytosis or ET. Some of these children were reported to harbor *MPL*^**S505A**^, which is actually *MPL*^**S505N**^, and in keeping with the original report in 2007.^21,22^ The precise significance and role of co-existing germline, driver and secondary somatic mutations in relation to age at the onset of the MPN clone are also poorly understood.^23^ Younger patients appear to develop PV following the acquisition of a single somatic *JAK2*^V617F^ mutation, in contrast to older patients in whom this mutation is often accompanied by secondary mutations.^24^ Interestingly, a diagnosis of MPN has been made in several children who were previously observed to have *JAK2*^*V617F*^ mutations detected in the newborn screening cards. Indeed, data from the Swedish-familial cancer registry suggest a genetic predisposition to acquiring MPN driver mutations at younger ages in patients with a high-risk family history. It is likely that inherited and environmental factors are shared in this genetic predisposition.^25^ Indeed, anecdotal reports of a *TERT* single-nucleotide variant has also been reported, especially in females, and associated with exposure to benzene and toluene.^26^

The International Pediatric Registry data suggest the incidence of CML in children and adolescents to be considerably lower than that in adults, with an annual incidence of about 0.7 per 1,000,000 and 1.2 per 1,000,000, respectively.^27^ The incidence of PV and ET has been estimated to be about 1 to 2 cases per 10,000,000 children and adolescents annually; the incidence of PMF and MF is unknown and considered extremely low.^28^ There are also some reports suggesting that with increased pediatric-MPN awareness, incidence rates could rise.

## Clinical and treatment issues in pediatric MPNs

Clinically, children and adolescents with CML in chronic phase often present with proliferative features, such as splenomegaly and higher leucocyte counts, compared with adults. Additionally, a greater proportion of them have advanced disease at diagnosis.^29^ Pediatric *BCR-ABL1*-negative MPN patients can present with thrombotic events, in particular when associated with JAK2 mutations.^30–32^ The risk of all thrombosis is quite low overall, estimated at about 9% for PV and 4% for ET. In contrast, the incidence of venous thrombotic events appears to be quite high – estimated at about 85% in the London series, whilst hemorrhagic complications are rare (<5%).^32^ Children with PV and ET also tend to exhibit higher white blood counts and often present with hepato-splenomegaly.^32^

For adult patients with CML, the licensed *ABL1*-tyrosine kinase inhibitors (TKI), accord an OS not dissimilar from that of the general population, and about one-half of patients achieving sustained deep molecular remission (DMR) are able to discontinue therapy successfully and durably.^1^ In contrast, though 3 TKIs are now approved for the treatment of CML in children and adolescent, the data with regards to efficacy, safety and treatment-discontinuation is considerably less robust, and uncertainty with regards to the optimal treatment remains.^5^ At present imatinib, nilotinib, and dasatinib are approved for first-line therapy. Imatinib has clearly improved clinical outcomes, and both nilotinib and dasatinib appear to achieve faster and deeper molecular responses, similar to those seen in adults. It is, however, unclear as to which TKI to offer initially, and no randomized studies comparing imatinib to nilotinib or dasatinib have been conducted so far. Neither are there are any specific ELN or NCCN guidelines for treatment or monitoring of children and adolescents with CML; rather the adult guidelines are often extrapolated.^27^ Compliance and adherence might be a bigger issue in adolescents and requires careful monitoring.

There is also considerable concern with regards to the long-term side effects of TKIs being administered during extended periods during growth and development, implying that treatment free remission (TFR) is an even more important goal than in adults. Further, the notion of TFR remains largely untested in pediatric populations, creating challenges in developing optimal treatment algorithms for children and adults with CML in chronic phase. A recent international study, STOP IMAPED, assessed imatinib discontinuation in 14 children with CML in chronic phase who had achieved and maintained DMR for >2 years.^33^ The study observed that the majority of study cohort experienced a molecular relapse following imatinib discontinuation with an overall probability of maintaining DMR at 6 months being 28.6%. In contrast to the recent EURO-SKI adult study, STOP IMAPED failed to reveal any predictive parameters, such as age, sex, time from diagnosis to the onset of therapy, treatment duration or duration of DMR until discontinuation, to be associated with molecular relapse.^34^ The precise reasons for these discordant results is unclear. It can be hypothesized that the presence of more proliferative features at diagnosis, different *BCR-ABL1* breakpoint distribution, and possible differences in the immune response between children and adults have contributed. Indeed, at present we know very little about the immunological mechanisms which may be relevant in maintaining DMR following TKI discontinuation. Regardless, further prospective and larger studies assessing discontinuing TKIs successfully are urgently required in children and adolescents with CML prior to the introduction of TFR into the pediatric clinics outside of clinical trials.

The recent approval of nilotinib and dasatinib for pediatric patients has also complicated the decision-making process regarding allogeneic stem transplantation (allo-SCT). Clearly, patients who have a suboptimal response or failure to 2 lines of TKIs should be considered for an allo-SCT. Allo-SCT should also be offered to CML patients who acquire the T315I mutation in *BCR-ABL1*, since the third generation TKI, ponatinib, is not currently licensed for children and adolescents. A recent comprehensive survey of 247 US-based pediatric hematologists’ attitude towards an optimal treatment algorithm suggest that most do not support the use of allo-SCT for first-line therapy, and about 40% support it if suitable donor was available.^35^ However, the vast majority support the use of allo-SCT following 2 lines of TKI therapy.

For adult patients with MF a qualified therapeutic success with a substantial relief of disease-related symptoms, splenomegaly and survival benefit has been observed with the only licensed JAK1/2 inhibitor, ruxolitinib,^36,37^ but therapy-related anemia is often an anticipated downside.^38^ Allo-SCT, however, remains the only treatment which can achieve long term remission and potential cure, but is associated with considerable morbidity and mortality.^39^ There are, however, scanty data with regards to the use of JAK inhibitors and allo-SCT in children and adolescents. Indeed, at present, there is no firm consensus on the optimal clinical management of children with PV, ET, or MF. Hydroxyurea is used frequently, but long-term safety remains unclear, and there is limited data available on use of interferon alpha in this patient population.^30,32^

## Future prospects

Arguably, despite much progress in the field of MPNs, and in particular CML, many important issues pertaining to the diagnosis, classification, risk stratification and treatment of children and adolescents remain. Mutational profiling has revealed important differences between adult and pediatric MPNs which influence the biology and prognosis, but larger contemporary cohorts with long-term follow-up are needed to assess this and the impact on treatment appropriately. In this regard, it is of note that during this year's International Childhood Cancer Awareness Day, both the Society for Pediatric Oncology Europe (SIOPE) and Childhood Cancer International (CCI) on Pediatric Hematology and Oncology launched a manifesto calling for the European Parliament to maintain and reinforce support. The rarity of pediatric MPNs and the increasing recognition of long-term side-effects of successful treatments, including cardiovascular, endocrine, growth and psychological issues underscore the urgent need for agreeable funding and collaborations worldwide.
